# Investigating Humor in Social Interaction in People With Intellectual Disabilities: A Systematic Review of the Literature

**DOI:** 10.3389/fpsyg.2018.01745

**Published:** 2018-09-21

**Authors:** Darren David Chadwick, Tracey Platt

**Affiliations:** Psychology, University of Wolverhampton, Wolverhampton, United Kingdom

**Keywords:** humor, learning disability, stigma, social support, developmental disabilities, autism spectrum disorders, social interaction

## Abstract

**Background:** Humor, both producing and appreciating, underpins positive social interactions. It acts as a facilitator of communication. There are clear links to wellbeing that go along with this form of social engagement. However, humor appears to be a seldom studied, cross-disciplinary area of investigation when applied to people with an intellectual disability. This review collates the current state of knowledge regarding the role of humor behavior in the social interactions of people with intellectual disabilities and their carers.

**Method:** A systematic review utilizing the Preferred Reporting Items for Systematic Reviews and Meta-Analyses (PRISMA) guidelines was completed, which aimed to explore the current state of knowledge and quality of empirical evidence relating to humor in people with intellectual disabilities. Following this, articles were grouped thematically and summarized. A comprehensive search of four electronic databases (1954–2017) and additional search strategies yielded 32 articles which met the final inclusion criteria.

**Results:** Humor played a significant positive and negative role in the social interactions of people with intellectual disabilities. Research had investigated humor in the classroom and humor expression in different groups including those with autism, Down syndrome, Angelman syndrome, Williams syndrome, and Rett syndrome. Few investigations directly studied humor appreciation and comprehension. Humor comprehension was reportedly supported by gestures. Some groups with intellectual disabilities found non-literal humor (e.g., sarcasm, irony) more difficult to understand, which may affect social relationships. Various types of humor were found to be appreciated. The role of humor in relationship development, social facilitation, creativity, and stigma had all received some limited attention. Humor also played a role for carer groups in coping with and enjoying the caring role. Research varied in quality with few experimental studies and mainly quasi-experimental and well-conducted, qualitative studies.

**Conclusions:** This review revealed the importance of humor behavior in many aspects of the social lives of people with intellectual disabilities. Limited disparate research exists pertaining to humor in this group, suggesting the need for further robust research in this area, including more high quality primary research in the areas of humor production, appreciation, comprehension, and stigma.

## Introduction

### Background and rationale

The population of people with Intellectual disabilities are extremely heterogeneous. They vary greatly in etiology, support needs, and comorbidities (e.g., health problems, mental health issues and physical, and sensory impairments). The clinical definition of intellectual disabilities provided by The World Health Organization (World Health Organisation, 1992) within the International Classification of Diseases (ICD-10) involves three criteria: (i) impaired cognitive functioning; (ii) Challenges to adaptive functioning in at least two key areas (i.e., Communication, self-care, domestic skills, social skills, self-direction, community, academic skills, work, leisure, health, and safety); and (iii) Early developmental onset (<18 years). Clinical definitions embody the medical model approach to intellectual disabilities (Chappell et al., 2001), which focus on individual differences and are primarily deficit and pathology focused, considering disability to be the product of the individual's impairments. However, more recent conceptualizations have highlighted that the purpose of identification of impairments and challenges faced by people with an intellectual disability is to identify necessary supports, which are typically provided by paid support staff or family carers. This is to help ensure that these people maximize their life chances, participation, and inclusion (Van Loon et al., 2010).

Nomenclature has varied across time, geographies, and cultures, with terminology often co-opted and naturalized within society as terms of derision (e.g., idiot, retard etc.), which serves to societally disempower, stigmatize, and devalue this group of people (Siperstein et al., 2010). Alternative social model perspectives focus on the ways in which societal, social and environmental factors disable people with cognitive impairments (Chappell et al., 2001). Thus, intellectual disability is also considered a socially constructed term, both historically and culturally bound. People are labeled as intellectually disabled because they differ from a culturally defined idea of “normal” or “typical” intellectual functioning (Manion and Bersani, 1987), facing societal disadvantage as a result.

Although there has been a concerted effort since the 1980s to remove social and physical barriers and moves toward equal citizenship and inclusion, individuals with intellectual disabilities still face numerous challenges in many aspects of their daily lives. From human rights issues, to experiencing the intolerance of others, they often face social, as well as physical exclusion (Amado et al., 2013). These issues can, and do, lead to social isolation (Abbott and McConkey, 2006). This exclusion extends into the world of research where areas studied extensively in the typically developing majority are often seldom touched upon in people with intellectual disabilities; the study of humor appears to be one such area. Moreover, exclusion may also occur due to perceived additional challenges and effort involved in identifying, classifying, and targeting those with an intellectual disability for study recruitment. Possibly due to the adaptations needed to enable people with differing support needs to participate. Hence this paper aims to collate and summarize the existing state of knowledge around humor in people with intellectual disabilities.

### The role of humor in lifestyle and wellbeing

There is evidence that eliciting positive emotions, such as fun and amusement are key components of positive social engagement. Therefore, it is also relevant for those with an intellectual disability. By its very nature, when spontaneous laughter, a non-verbal vocalized expressive communication signal of amusement occurs, it alters the state of consciousness and allows for “*care, trouble, and even physical pain”* to be forgotten (Hall and Allin, 1897, p. 8). One way that spontaneous laughter can be elicited is through humor. A myriad of situations can be deemed humorous. Humor appreciation goes along with individual differences but falls into three main areas, non-sense, incongruity resolution, or sexual (Ruch, 1992). Most individuals will find some aspects of such situations, or jokes, funny. When we share or engage others, in humorous situations, it serves a number of social functions. For example, Brown and Levinson (1987) suggest that jokes are positive politeness strategies for minimizing face threatening situations. Further evidence of the social function of humor in interpersonal relationships has been demonstrated by Holmes (2006), who showed that within the workplace, humor fosters collegiality and is also used to both construct and maintain good relations. As many people with intellectual disabilities require support outside of the inner family circle (MacTavish et al., 2007), there is a need for a better understanding of the role that humor plays within different relationships.

However, in order to fully understand this dynamic, one also needs to consider that individual differences will play a role. Being high in trait and state cheerfulness, low in seriousness and bad-mood relates to the temperamental basis for a sense of humor (Ruch et al., 1996). Those high in either state or trait cheerfulness will more readily be influenced by exhilarants (stimuli that elicits laughter and amusement)—one of those being the propensity to engage in humor. Cheerfulness has been shown to correlate negatively with both seriousness and bad mood, whereas seriousness and bad mood positively correlate (Ruch, 1997). Being cheerful allows for a lower threshold for engaging in humorous behaviors and finding things amusing. Clearly, cheerfulness relates to positive affect and extroversion (Ruch, 1995), and thus those with the propensity to be cheerful and engage in humorous behaviors, are those we orientate toward.

As well as being linked to aspects of relationship building and maintaining, humor directly links to positive affect and enhanced quality of life (Kuiper et al., 1992). Consistently, positive affect has been shown to be associated with good health, well-being, and with health protective responses (Pressman and Cohen, 2005). Fredrickson (2000) suggested that the cultivating positive affect can optimizes health and well-being which has the potential to be induced or elicited by, among other things, humor (Baron et al., 1990).

### Defining humor

Moran (2013) noted “humor” is a term with a multitude of meanings. That it is both a “cognitive style”; a term for a stimulus, as well as the response to it (e.g., laughter). She also stated that humor is a term relating to complex interactions between individuals, or for a broader social process; a “personality trait,” or an inherent characteristic; an ability to generate a response, produce a response, or detect/observe the two. Finally, she added that the complexity was compounded by the notion of “comedy” which has its own set of interpretations and expectations.

When discussing the positive benefits of humor in all aspects of social interactions it is essential to define this complex construct, as many theories exist and not all may have the functions being discussed in this paper. For example, laughing at someone, or mocking them, is a form of humor interaction but will neither foster good relationships nor elicit positive affect in the target of that mockery. However, teasing, which also relates to “play” laughing at a target, serves a pro-social function, and even seen as part of flirting behavior (Keltner et al., 2001). So how humor is perceived, will depend on the both the context, and the disposition of the actors within the interaction.

One classification was proposed by Schmidt-Hidding (1963) (see Ruch et al., 2017 this volume for an overview). These eight styles of the comic: *humor, fun, nonsense, wit, irony, satire, sarcasm*, and *cynicism* are a useful way of determining the differences. It should be noted in this context that humor is unique from the seven other comic styles and is classified as “coming from the heart” (see Table 1).

**Table 1:** Schmidt-Hidding comic style for humor.

**Characteristics**	**Humor**
Intention, Goal	To arouse sympathy and an understanding for the incongruities of life
Object	Creation in all its forms; human and real issues
Attitude of the agent as subject	Distant, affirmative, conciliatory, tolerant, love of the individual creation
Behavior toward the next	Understanding, benignly including oneself in judgments
The ideal audience	Jovial, relaxed, contemplative
Method	Realistic observation
Linguistic peculiarities	Ambiguous, without punch line; first-person Narration preferred; dialects, and professional jargon

Table 1 shows how Schmidt-Hidding defined humor. The goal of this definition of humor is to raise our understanding of the incongruities of life, while remaining sympathetic for the human condition. This form of humor holds an understanding for the other, and any humorous judgment will benignly include oneself, rather than maliciously being directed at a target (e.g., when laughing at). The opposing dimension of this benevolent humor would be ridicule or mockery (see Ruch et al. this issue), where those deemed as being weaker or as being from an out-group become the object or target of derision and this fine-grained definition of the forms of humor would be important when investigating humorous interactions of and with people with intellectual disabilities.

### Humor and intellectual disability

Little is known about humor in relation to people with intellectual disabilities. The development of the sense of humor is well established and broadly depends on cognitive, social, and individual difference variables. For verbal humor, such as joking, a greater cognitive capacity is required (McGhee, 1979), for example. McGhee (1980), also found that humor develops, from among other things, physical and verbal assertiveness and dominance. Due to the cognitive impairments which characterize intellectual disabilities, it is probable that people with intellectual disabilities may experience challenges in cognitively processing, comprehending, and appreciating humor. Moreover, physical and assertive dominance is likely to be more limited due to the limitations in self-determination, autonomy, and expressive communication.

As the participation in humorous interactions requires both en/and decoding of the play signals, associated craniofacial differences may affect the expressed enjoyment, which may be prohibitive of sustained interactions where humor is exchanged. Conversely, the genetic condition Angelman syndrome includes, as part of its behavioral phenotype, frequent expressions of smiling, and laughter. Though not always the case (see Oliver et al., 2002), these facial and vocalized expressions being displayed may simply occur when no stimulus is present (Nirenberg, 1991) or be disassociated from the context (Bower and Jeavons, 1967). Therefore, breeching the rules of communication that make interactions more difficult to establish and maintain.

### Objective

This review aims to investigate the state-of-the-art in the existing empirical evidence regarding the interactional and experiential aspects of humor for people with intellectual disabilities, and those who support them. To this end a systematic review was conducted of the extant literature to address the following questions.

#### Research questions

1. In what ways has humor and laughter been empirically explored and investigated in the lives of people with intellectual disabilities?
2. Is humor behavior a significant component of the social interaction of people with intellectual disabilities?
3. What is the quality of empirical evidence regarding humor in people with intellectual disabilities?

## Method

### Study design

This systematic review study is underpinned by transformative and positive psychology epistemological perspectives, aiming to provide knowledge which can be used to improve the lives of people with intellectual disabilities. It collates and synthesizes literature underpinned by postpositivist, phenomenological, and constructivist epistemologies. From this framework, it aimed to highlight the emergent themes around humor interactions and the experiences of people with intellectual disabilities and their interaction partners (e.g., carers and family members). We predict that humor will play an important role in the social interactions of people with intellectual disabilities.

### Systematic review protocol

This systematic review employed the Preferred Reporting Items for Systematic Reviews and Meta-Analyses (PRISMA) (Moher et al., 2009) as a guide to help ensure rigor. Also, a 5-step approach was utilized in implementing the systematic review as outlined by Khan et al. (2003) as follows:

#### Framing questions

From the existing expertise of the authors and an initial scoping perusal of the extant literature it appeared that literature focusing on humor and people with intellectual disabilities was scant.

#### Identifying relevant publications

##### Search strategy

For this literature review a search in the Web of Science (SCI-EXPANDED, SSCI, and A and HCI) and EBSCO (British Education Index, Child Development and Adolescent Studies, Cinahl, Education Research Complete, ERIC, Humanities International Complete, Medline, Psychology, and behavioral sciences collection, PsycINFO and SocINDEX) databases was conducted in April 2017 (Search dates ranged between 1954 until 2017) and subsequently updated in September 2017. All English language papers containing the terms “Intellectual disability” or “learning disability” and “humor” or “humor” with the searches combined terms for humor and intellectual disabilities with the Boolean operator “and” in the title or abstract were identified (Note: the search engines also identified and included related terms in the searches). An example of database specific search terms (Psychinfo) is given in Appendix 1.

**Figure 1:**
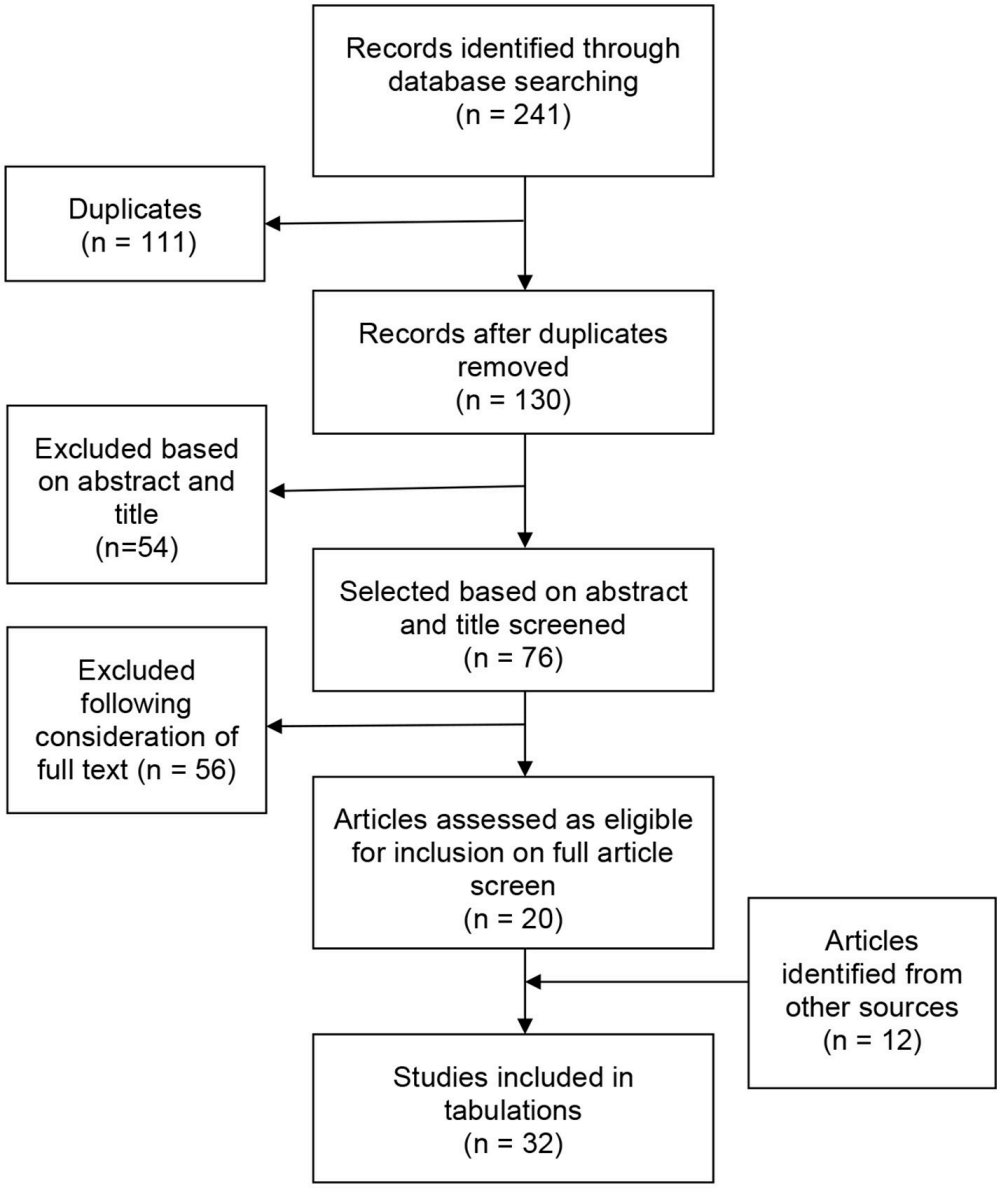
Flowchart of study identification.

The titles of these studies (see Figure 1) were then inspected to ascertain whether they were likely to contain information, which could aid in answering the questions developed for this review. Once a primary list of articles had been identified a secondary review of the title and abstracts was conducted. Full texts were then gathered and reviewed for inclusion (see below for criteria). Reference lists of these identified studies which met the inclusion criteria (see below) were searched to identify further papers for inclusion. Full texts of salient articles identified this way were then gathered and full reviews conducted for inclusion.

In addition, in March 2017 a request for information on research relevant to humor and people with ID was sent to members of the International Association for the Scientific Study of Intellectual and Developmental Disabilities (IASSIDD) Quality of Life Special Interest Research Group and the Intellectual Disability UK Research mailing list, with the request subsequently being published in the TAC Bulletin in October 2015 (www.teamaroundthechild.com). Furthermore, the same enquiry was made to the International Society for Humor Studies (ISHS) members as well as listserve questions asking for relevant information. Finally, a paper presentation was given at the Annual ISHS conference in Montreal (July, 2017) where requests for information on relevant papers was made.

The authors subsequently identified and reviewed English language studies, focusing on humor interactions by people with intellectual disabilities. Contextually and due to the literature gathered, this paper is written from a UK perspective, but also incorporates research from North America, Asia, Australasia, and other parts of Europe (see Appendix 2).

##### Inclusion criteria

Studies were required to meet all of the following criteria: Collection of empirical data; peer reviewed; English language full text; published between 1950 and 2017. Inclusion criteria germane to the focus of the review were as follows:

- Studies included had to include as participants or be focused upon people with intellectual disabilities and/or those with developmental disabilities where intellectual disability is a core component e.g., people with Autistic Spectrum Disorder (autism), Rett syndrome.
- Core papers had to focus on humor and laughter in terms of either: (i) it being the primary focus of the paper; or (ii) it being a key finding from the empirical work.
- Particular attention was given to studies investigating or presenting findings which centered on the interactional components of humor in the lives of people with intellectual disabilities.

As we were also interested in how humor had been conceptualized and studied in the lives of people with intellectual disabilities, we also included some papers outside of these inclusion criteria which focused on analysis of secondary data in an area of study considered important to the lives of people with intellectual disabilities but seldom investigated in terms of primary data (i.e., the relationship between humor and stigma) or focused on carers and professionals who supported people with intellectual disabilities.

##### Exclusion criteria

The following exclusion criteria were applied: not peer reviewed or where the peer review status was deemed unclear; reviews, letters, commentaries, editorials, meeting, or conference abstracts; study relates solely to infants (less than 1 year of age). Those articles that did not relate sufficiently to either humor or intellectual disabilities were excluded. We also excluded papers which focused on people with developmental disabilities where intellectual disabilities are not a principal component (i.e., specific developmental disorders, attention deficit hyperactivity disorder, Asperger syndrome etc.).

Following secondary screening by title and abstract, we included two new exclusion criteria. First, any article that focused purely on phenotypic aspects where humor was not a central consideration but a description associated with the phenotype. Second, papers where the focus is on fun and enjoyment as ways of eliciting engagement rather than specifically focusing on interactional and experiential aspects of humor.

#### Summarizing the evidence

The findings were summarized in two key ways. Firstly, tabulation of the papers pertinent to humor in people with intellectual disabilities that help shed light on main areas of research. This was supplemented by a thematic organization of the papers which developed from the extraction of data on the foci and findings from the studies in accordance with the specified research questions addressed. Meta-analysis was precluded by heterogeneity across studies.

#### Assessing study quality

Critical appraisal of the quality of the studies and risk of bias for the retained articles was conducted using the QualSyst quality appraisal tool for quantitative studies (Kmet et al., 2004) to allow comparability across studies. Authors independently generated quality scores of “yes,” “no” or “partially” for each article on each quality indicator (14 for quantitative and 10 for qualitative studies). To ensure replicability and objectivity, the goals of this systematic and conceptual review were registered on PROSPERO the International prospective register of systematic reviews, prior to the research being conducted (https://www.crd.york.ac.uk/PROSPERO/), registration number CRD42017070222.

#### Interpreting the findings

Finally, based on the number and quality of studies reviewed, conclusions were drawn in relation to the questions posed at the outset of the review.

## Results

### Summary of included papers

Electronic database searches identified 241 references, with 214 remaining after removal of 27 duplicates. Following an initial screen of the peer-reviewed papers based on the paper title and abstract, 138 references were excluded with 76 remaining for further screening. After examination of full text and the addition of studies cited within these, 32 studies met the inclusion criteria. These are summarized in Appendix 2. A flow diagram of the process undertaken is presented in Figure 1.

Of the 29 papers that met the inclusion criteria 13 were qualitative, 19 quantitative. With regard to the methodology employed within the qualitative papers: Two papers involved description of educational or mentoring programs, with only one of these qualitatively evaluating the program; Four studies used face-to-face interviews with either carers (*N* = 3), or people with intellectual disabilities (*N* = 1); Four conducted qualitative analysis of observational interactional data; finally, three papers, pertaining to humorous media representations of disability, involved analysis of comments on media or analysis, and reflection on film portrayals of intellectual disability. One of the papers (Johnson et al., 2012) analyzed both observational and interview data. Two of the qualitative papers considered humor specifically within online contexts (YouTube/Facebook).

Considering the methodologies used within the quantitative investigations: Three were descriptive studies using survey or observational methods, a fourth descriptive study used a cross-sectional design gathering data using specifically devised materials to ascertain comprehension/appreciation of humor; Two were longitudinal cohort studies. In addition, many studies employed quasi-experimental approaches (*N* = 11): One was a case series utilizing an ABAB design; Ten were comparative ex-post facto design studies taking either cohort (*N* = 5) or case-control (*N* = 7, two studies included both elements) approaches. The first considered potential differences across different cohorts with intellectual disabilities (i.e., People with autism, Down syndrome, Williams syndrome etc.), whilst the case-control studies compared people with intellectual disabilities to typically developing controls, who were often matched on age; Finally, two were true experiments, being small scale, within group studies of humor expression in people with Angleman syndrome observed under different interactional conditions.

With respect to the different subgroups of people with intellectual disabilities recruited into the studies, many (*N* = 18), unsurprisingly, had no specified etiology or diagnosis reported, with six papers being unclear about the extent to which people with intellectual disabilities were included in the study participant group. More specificity was evident in some papers (*N* = 12) with studies including people with autism (*N* = 5), Down syndrome (*N* = 5), Angelman syndrome (*N* = 3), Williams syndrome (*N* = 3), Prader Willi syndrome (*N* = 1), and Rett syndrome (*N* = 1) all encountered in the review, with some studies (*N* = 6) focusing on more than one group. Finally, paid and family caregivers or educationalists as key stakeholders in the social lives of people with intellectual disabilities were key participants in nine of the studies. With regard to the level of intellectual disabilities of participants in the studies, again for over a third of studies (*N* = 12) this was not specified, with roughly equivalent numbers focusing on people with borderline/mild (*N* = 8), moderate (*N* = 8), severe (*N* = 6), and profound (*N* = 8) intellectual disabilities. Again, many studies (*N* = 8) included people with different levels of intellectual disability. Finally, almost two-thirds of the studies (*N* = 18) focused on children and adolescents, with 11 papers focusing on adults, two papers spanned both age groupings and four studies did not specify the age group of the participants.

### Synthesized findings

Eight themes were determined from the post-scrutinized papers. Humor was studied in different and competing ways in the identified literature. To facilitate interpretation of data, findings are organized and presented by these emergent themes.

#### Humor comprehension and appreciation among people with intellectual disabilities

It emerged that five studies explored humor comprehension and preferences in people with intellectual disabilities. This supports the notion that this important communication behavior is a neglected area of study in adults with these disabilities, requiring further investigation. Findings suggest that young people with intellectual disabilities show appreciation of humor (Degabriele and Walsh, 2010). However, humor comprehension was poorer in people with DS and WS compared with age matched to typically developing controls (Krishan et al., 2017). The authors suggest no association between humor appreciation and theory of mind in these participants. Difficulties in social problem-solving and incongruity understanding may impede humor comprehension in children with intellectual disabilities (Short et al., 1993). There is a tentatively indication that comprehension of non-literal humor, including irony and sarcasm, might be reduced in people with Williams syndrome (Godbee and Porter, 2013). This may be due to differential development of linguistic and cognitive systems, which may impact on their social interactions and relationships. Ironic jokes were misclassified as lies by adolescents with Williams syndrome and Prader Willi syndrome (Sullivan et al., 2003). With regard to support for humor comprehension, gestures may potentially be a useful support for humor comprehension in young people with intellectual disabilities (Degabriele and Walsh, 2010).

Degabriele and Walsh (2010) investigated the development of humor [appreciation (and) comprehension] in nine children with intellectual disabilities aged between 7 and 11 in the Republic of Ireland. Participants with intellectual disabilities rated short video cartoon scenes and found that physical (85%) and visual (84%) humor scenes were more greatly appreciated by school aged children with intellectual disabilities compared with non-specific scene from a cartoon where no humor was evident (74%). Verbal humor was not appreciated significantly more than the non-specific scenes. The non-specific scene in the cartoon were highly appreciated too. Degabriele and Walsh (2010) in their study also investigated comprehension of humor and found that jokes supported by gestures (rather than pictures or acting) were significantly more understood by the young people with intellectual disabilities. Phonological jokes were best understood by participants but other joke forms (lexical, syntactic, and semantic) were also understood.

One study by Short et al. (1993) analyzed the humor skills of elementary school students, investigating those with and without intellectual disabilities on the dimensions of humor comprehension, but also included production and appreciation. This rather early study used common American terminology for their groups, which would not be understood world-wide. For example, they had an achieving normally group, a group with learning disabilities and a group with developmental handicaps. The group with learning disabilities often showed no difference to the achieving normally group and this is most likely due to this group included children with IQ >85. The children with developmental disabilities lacked differential sensitivities to cartoons, which the authors suggest is down to their social problem-solving deficiencies or ability to represent the problem to understand the incongruity and the process of resolution. However, the authors did not take other forms of humor appreciation (e.g., sexual/scatological and non-sense humor) into consideration in their conclusions, which limits this more comprehensive and insightful study.

Utilizing a comparative study to investigate aspects of humor comprehension and its connection to aspects of Theory of Mind, Sullivan et al. (2003) had groups of adolescents, one with William syndrome, another with Prader-Willi syndrome, and a group which had non-specific intellectual disabilities try to distinguish between different forms of non-literal language used in stories that ended in either a lie, or an ironic joke. To do this, the authors manipulated the structural differences in the child's second-order belief about the adult's knowledge of the truth of the situation. This research found that almost none of the participants in any of the groups were able to correctly classify the ironic jokes, instead judging them to be lies because they did not correspond to reality. Their errors were similar to those made by younger normally developing children, but contrasted with those made by brain-damaged adults. The authors state that the consequences of this inability to distinguish between intentionally false utterances, intended as ironic jokes vs. those intended to deceive, may seriously impairs these adolescent's ability to relate to others in everyday social situations.

A similar study by Godbee and Porter (2013) pursued two aims in their study. They aimed to investigate the comprehension of sarcasm, metaphor and simile in people with Williams syndrome compared to neuro-typical controls, secondarily, they aimed to examine the association between non-literal language comprehension and a range of other cognitive abilities, both in Williams syndrome and in the neuro-typical population. Matching both chronological and mental aged groups, all participants listened to randomly selected stories. After each comment from a story character, the participant was asked what the character meant by their comment. The comments were coded for whether the reply demonstrated correct understanding of the non-literal meaning of the comment; otherwise, they were given a zero score. Several types of responses were awarded a score of 0, including: literal explanation; ambiguous explanation; irrelevant explanation; no explanation; recognition of non-literal language without interpretation (e.g., he doesn't mean it); and supply of another non-literal comment without interpretation. For the comprehension of non-literal language, the individuals with Williams syndrome performed significantly below typically developing chronological age matched controls. However, they did not demonstrate significant differences to typically developing mental age matched controls. For the typically developing controls, each of the cognitive measures was strongly correlated with each of the measures of non-literal language comprehension. The same relationships were not always found for participants with William syndrome. In particular, sarcasm comprehension in participants with William syndrome was not significantly correlated with any of the assessed cognitive abilities. The expressive vocabulary was not significantly correlated with any measure of non-literal comprehension. The pattern of correlations between non-literal comprehension and cognitive abilities in the group with WS, relative to the control group suggests that perhaps the linguistic and cognitive systems that underpin non-literal language comprehension in neuro-typically developing individuals interact and integrate in different ways to individuals with Williams syndrome.

A further study conducted by Krishan et al. (2017) investigated humor comprehension and use of mental state language in groups of individuals with Williams syndrome and Down Syndrome relative to each other and to a neuro-typical control group. These groups were chosen for the link of humor to Theory of Mind (ToM) to fill the gap in the literature which focuses on those with ToM deficits such as those with autism. Relative to the control group, both groups of participants with intellectual disabilities had poor humor comprehension. The William Syndrome and Down Syndrome groups had comparable performance to each other, as well as to a mental age matched control group, differing only in physical emotion words, where those with William Syndrome used fewer. The use of cognitive words was less for both groups with intellectual disabilities. The authors also suggest that humor appreciation is not associated with theory of mind in people with Williams syndrome and Down syndrome.

#### Humor, social facilitation and social capital

Studies reported findings where humorous exchanges, in particular banter and sharing of humor, were identified as significant, enjoyable components in the facilitation, development and maintenance of social relationships, and capital. They also identified how humor served to enhance social closeness facilitating intimate shared connection between people with intellectual disabilities and those supporting them. Attunement of those providing support to those with more significant cognitive impairments was highlighted as positive components of social interaction, including attuning of the type of humor (e.g., slapstick).

Griffiths and Smith (2016) aimed to identify the process that regulates communications of people with profound and multiple learning disabilities (PMLD) with others. They used fine grained (second-by-second and frame-by-frame) qualitative analysis of video-recorded observational data from two dyads of people with PMLD and carers, in a developmental disability center for young adults in Ireland. Glasserian grounded theory was the analytic approach used to develop a theory of attuning. This theory asserts that communication takes place in the context of a physical setting. The setting influences the state of mind of those within the interaction. In turn, this influences the stimuli they present which may or may not be attended to by the communication partner. Attending to stimuli is also affected by the setting in which the interaction takes place. Engagement occurs when one player attends to the stimuli of another, the determining factor is the process of attuning. Attuning affects and reflects the feeling of the communication partner in terms of whether they offer stimulus to their partner, attend to the other, engage with the other and act. All of these processes feedback to each communication partner to influence their state of mind (being). Thus, attuning is an implicit, cognitive process that is not observable in of itself, but there are behaviors which are observable and which indicate attuning is taking place. Here, humor is evident in the example data used to illustrate the theory. Humor is described as an indicator of empathic harmony and pro attuning and a manifestation of: (i) close psychological contact via a smile; (ii) shared amusement via a smile or laughter. In a sister paper focusing on the same data set, Griffiths and Smith (2017) briefly mention joking as an exemplar of solidarity in a group situation which could foster an intense level of attuning between people with PMLD and their carers. Although this evidence may seem less substantial, it is a good indicator of the importance of how this form of humorous banter facilitates in-group cohesion.

Johnson et al. (2012) similarly studied the lives of six people with severe intellectual disability, with symbolic but non-linguistic communication skills, and their interactions with others. In this Australian study, they observed interactions between people with severe intellectual disabilities and others and interviewed interaction partners and again analyzed via constructivist grounded theory. Social interactions took place when dyads and groups “shared the moment” this central theme was characterized by hanging out and having fun together. The latter of these involved both routines, utilizing activities such as mimicry, rhythmic play, games, songs, and comedy. Comedic interactions observed comprised several different forms of humor including vulgarity, pranks, jests, and banter. The exert of involvement and initiation differed both across the types of humor (banter occurring more often between support staff but involving people with intellectual disabilities) and participants (three participants were observed to initiate humor, whilst the other three adopted the role of active respondents and joined in with humorous interactions). More vulgar humor was sometimes supported and encouraged and other times discouraged. The humorous interactions were described as animating and enjoyable for the parties participating, fostering a sense of belonging. It is hypothesized within the paper that visual humor (i.e., slapstick) may be enjoyed more by participants because it relies less on verbal skills. Teasing was also observed and was noted to be used by familiar staff to improve the mood of people with severe intellectual disabilities.

Chadwick and Fullwood (2017) conducted a small UK and Ireland based qualitative, phenomenologically focused, study of the online lives of eleven people with mild to moderate intellectual disabilities. Two had Down syndrome and five had autism. They identified two global themes around the online lives of these participants (i) Online relatedness and sharing; (ii) Online agency and support. For the former theme, one basic theme 'coming together on social media with friends and family to chat and share' related to sharing online life and being connected to significant others which supported maintenance and development of social capital with family and friends. One important component of these interactions referred to by four of the eleven participants was humor, which took the forms of playing practical jokes, banter, and 'taking the Mick out of each other' and these interactions were viewed positively by participants as the most enjoyable online activities they engaged in.

#### Classroom humor and laughter

Four papers focused on humor in the classroom and one on changing behavior in pre-school children. Schnitzer et al. (2007) investigated the Feuerstein's Instrumental Enrichment Program (FIEP) as a means of increasing social, cognitive function. Here the comprehension of humor, even complex humor, was one goal of the experimental group who had the FIEP intervention. As part of the GOLD program, designed to support children who were gifted (defined as having IQ potential determined by a screening committee) and also had intellectual and/or developmental disabilities, Bees (1998) highlighted that humor was encouraged and having time for laughter was a way of helping the children relax. These papers highlight the conflicting perceptions of laughter and humor within the classroom context. Unabashed, shrill laughter, was not a welcomed behavior, yet prescribed moments of humor and laughter were seen as beneficial. However, laughter and humor are, by their very nature, organic and as beneficial as allowing for moments of hilarity are, maybe these benefits flourish more when not so prescribed? This idea was reiterated by the study of Jones and Goble (2012), who investigated effective campus mentors in partnerships with students with intellectual disabilities. They identified the key components for effective mentoring partnerships. One of those was of prioritizing fun and socializing, which, they suggest, should happen spontaneously. An afterschool program was designed to enhance character trait development. It utilized high school and college mentors to both introduce the program's curriculum and to help build friendships (Muscott and O'Brien, 1999). This component was key to the program's success, as the outcome was that the children with intellectual disabilities had found that learning about character was fun and the program rewarding.

The play behaviors of school age children with intellectual disabilities were assessed by the observational Assessment of Ludic Behavior instrument which measured three dimensions: play interests, play abilities and play attitude (Messier et al., 2008). The findings of this study showed that the sense of humor (as well as enjoyment of challenge) were less present than other elements of the test. A component of the ludic attitude dimension, the sense of humor factor, was scored when the child was deemed to show a sense of humor, an understanding of comical situations, and laughs. The authors argue that this deficit is due to humor requiring a complex cognitive ability. Yet, they also raised the point that studies have shown these are often in conflict to parent's observations, who reveal higher scores than therapists do. This demonstrates the benefits of having mentors who build friendships and those close parental ties to the children, as they can often better see and attribute the subtle differences.

#### Humor and creativity

People with intellectual disabilities have been shown to be creative in their humor use (Johnson et al., 2012). People with autism and intellectual disabilities, who have been found to display less playful pretending (Hobson et al., 2009), have demonstrated the ability, with prompting, to enhance their humorous creativity (Gagić et al., 2015).

Johnson et al. (2012) discussed the issues around the language skills of adults with severe intellectual disability and how they are limited and this impacted on the range of humorous forms. However, they found that the participants of their study did demonstrate “creativity and variety” (pp. 338) in their attempts at humorous social interaction.

This creative use of humor in social interaction was very different for those children with autism, for example. Hobson et al. (2009), measured both spontaneous and modeled symbolic play, in those with and without autism. They predicted that play for children with autism would lack social-developmental markers. Speculating that this form of play with an investment in the symbolic meanings given to play materials, creativity, and fun. They found that children with autism displayed less playful pretending and investing in symbolic meaning of the items given to play with. However, the study did not have ratings for the produced observed creativity with the play, which would be required, given the low expressivity of children with autism.

(Gagić et al., 2015) used humorous content as an indicator of the expression of creative ability in a drawing task. They used a method of prompting to encourage creative thinking around the art and showed an increase in the humor within the work, post prompting. This kind of prompting and engagement with play may be a way of engaging those who seems to be limited in the social aspects of creative play, such as children with autism.

#### Play, humor and laughter in children with autism and down syndrome

Some of the identified papers and themes, focused on specific groups of people with intellectual disabilities associated with specific syndromes and how humor is understood, expressed and used in these groups. Diagnoses including Autism, Down syndrome, Angelman syndrome, Williams syndrome, Prader Willi syndrome and Rett syndrome were studied, here we collate research focusing on the first two of these groups.

Four papers investigated the play, the humor and laughter of children with autism and, in one instance, compared them with children with Down syndrome. Hobson et al. (2009) testing pretend play abilities in children with autism and children with learning and developmental delays but without autism, found that although both groups were similar in the mechanics of play, the children with autism showed lesser qualities of playful pretend meaning the awareness of self as creating meanings, investment in symbolic meanings, creativity, and fun. Although this paper focuses on the deficits relating to autism, conversely it highlights that the children with the intellectual disabilities in this sample do not lack these qualities of play.

Reddy et al. (2002), interviewed parents who reported on specific incidents relating to their child's humor. Interview questions focusing to the type of things the child normally finds funny or laughs at, the attempts to join in with others' laughter, repeating others' laugh events (clowning), and teasing by the child or parent were compared in a group of children with autism and a matching group with Down syndrome. Significant differences were found that the majority of parents of children with Down syndrome reported their child tried to join in when others are laughing, whereas only five of the 18 children with autism had such behavior noted by their parents. Similar differences were reported for trying to make others laugh and teasing conditions. Group differences were observed by coding laughter episodes of videoed play sessions. No group differences were found in the frequency of laughter episodes or the rate per hour of laughter started by the children or in interactive situations. This study highlighted that the children with Down syndrome displayed all typical infant development of humor whereas the children with autism only showed some aspects.

Focusing on the vocal expressions of laughter, produced by children with and without autism, Hudenko et al. (2009) recorded laughter during play involving age appropriate humor stimuli that was based on ideas of humor development by McGhee (1979). The children with autism only exhibited one type of laughter compared to the comparison group, who produced two types. Other variables (fundamental frequency, duration, and number of laugh bouts etc.) did not show group differences. The authors argue that their findings indicate that the laughter of children with autism are responses to internal positive states, whereas those children without autism also utilize laughter to negotiate social interactions.

The remaining study was conducted by St. James and Tager-Flusberg (1994). They investigated the cognitive developmental, social and intentional aspects of naturalistic humor in two groups of six children, one with autism and the other Down syndrome. The children were filmed when interacting with their mothers in twice monthly, 1 h long, video-taped sessions. The authors report that the group of children with autism produced less humor overall and less humor that involved non-verbal incongruity. The only two jokes observed were created by children with Down syndrome. As with the other studies, deficits in the social-cognitive aspects of humor were highlighted for the children with autism.

#### Laughter as disruptive, unelicited, or inappropriate social behavior

In addition to being a means of facilitating social closeness, supporting learning and creativity, research had also focused on laughter as an unwanted, disruptive, unelicited and/or inappropriate, social behavior. Some studies focused on reducing such behavior via corrective intervention, others investigated the trajectory of unwanted laughter as people age, whist other considered whether laughing behavior was unelicited or a response to social and environmental stimuli.

#### Reducing disruptive laughter

A paper by Schieltz et al. (2011) investigated a dedicated program which was designed to target disruptive social behavior in pre-school children. Schieltz and colleagues evaluated functional communication training as a means of correcting destructive and disruptive behaviors, one of the non-targeted disruptive behaviors was *shrill laughter*. Despite the lack of targeting post intervention all undesirable behaviors, including the shrill laughter, reduced.

#### Night laughing in people with rett syndrome

Rett syndrome is a rare neurodevelopmental disorder which usually affects females. It is associated with a mutation in the MECP2 gene (Amir et al., 1999). Sleep problems have been noted as common in this group and are incorporated into the diagnostic criteria (Kaufmann et al., 2010). These problems manifest as night laughing or night screaming in young children (Hagberg, 2005) and linked to immature sleep patterns (Nomura, 2005) and can negative affect parental relationships and social activities (McDougall et al., 2005).

Wong et al. (2015) studied sleep disorders in this group in Australia in a longitudinal cohort study gathering data at 6 time points over 12 years. They found that more than 80 per cent had sleep problems, but prevalence decreased with increasing age. Night laughing was frequently evident. It occurred in 77 per cent when younger and those with a larger gene deletion had higher prevalence of night laughing. They found that behavioral and pharmacological treatments were associated with a 1.7 per cent reduction in risk of further sleep problems.

#### Laughter in people with angelman syndrome

Angelman syndrome occurs in 1 in 10–12,000 live births and is associated with various degrees of intellectual disabilities (though typically severe to profound cognitive impairment) and greater impairment of expressive over receptive speech (Steffenburg et al., 1996). Physical signs of Angleman syndrome include ataxic gait, craniofacial differences, hand flapping, and hypopigmentation. The behavioral phenotype includes elevated levels of smiling and laughing (Adams et al., 2015), with early studies describing smiling and laughing in this population as excessive and occurring without stimuli. A body of research work has been conducted by Oliver and associates incorporating humor related behaviors (Laughing/smiling) and exploring the role of social and environmental influences on these behaviors. Due to the rarity of this condition these investigations involved small numbers of participants.

Oliver et al. (2002) in a case series of three people with Angelman syndrome living in the UK and Greece found that smiling and laughing was greatest when enthusiastic interaction was taking place, moderate in instructional interactions and when there were others present but no interaction (proximity condition), and lowest when individuals were alone. This finding disputes the earlier assertion that smiling is inappropriate and is not elicited by environmental stimuli indicating a social function for these behaviors and an interaction between the phenotype and environment.

In 2015 Adams et al. published a brief report on a longitudinal UK based study of laughing and smiling in 12 young people with Angelman syndrome across full interactional (with eye contact), interactional (without eye contact) and proximity conditions. The findings revealed that smiling and laughing reduced with age during full interactions for participants as they move from childhood into/toward puberty/adolescence. Thus, an interaction between behavioral phenotype, environment and aging is apparent from the data. The need to explore further how puberty affects physical, emotional, and social development in people with intellectual disabilities is highlighted here.

Mount et al. (2011) in a study of the effects of familiarity and eye contact on the social behaviors of people with Angelman syndrome found that although they were the most variable social behaviors observed, more laughing/smiling was observed with familiar contacts when eye contact was maintained, though this finding did not reach statistical significance, likely due to the small sample size (*N* = 15) in the study.

#### Humor as a coping strategy for carers and support staff

One of the ways in which humor and shared humor operated as important aspects of the social worlds of people with intellectual disabilities was as a coping strategy carers used to manage and bring enjoyment and value to the caring responsibilities and societal stigma which accompanied their role. This was found in three of the identified articles.

MacDonald et al. (2007), in a cross-sectional descriptive survey study of respite care and coping strategies employed by family carers in Ireland, found that over 80 per cent of both male (81.5%) and female (81.8%) carers reported that ‘seeing the funny side of the situation' was employed as a managing meaning coping strategy. Such strategies were frequently employed by carers to enable them to maintain a sense of humor regarding their role. It also reportedly supported them to remind themselves that the person with a learning disability who they supported, was not to blame for their behavior and support needs.

In a qualitative interview based study with eight paid staff members working on a treatment program for sex offenders with intellectual disabilities, Sandhu et al. (2012) investigated the emotional challenges these staff faced. Interpretive phenomenological analysis revealed that humor was, once again, used as a way of dealing with negative emotions arising from working in this context and with this group of people. Banter and a “sick” sense of humor reportedly helped staff to process negative emotions that they otherwise may carry with them. There was also a sense of sharing and bonding over this “sick” sense of humor that was seemingly viewed by respondents as exclusive to colleagues working in this field. In addition to being a coping strategy to help staff process the stress of work, humor was also interpreted as a defense mechanism which prevented the staff team from exploring the personal and emotional impact of work. The authors viewed this as having potentially negative consequences for the wellbeing of staff and the therapeutic process for clients. Another feature of the narratives from staff was that empathy for the people with intellectual disabilities that they worked with was challenging and complex due to their emotional responses to the offending behavior.

Forster and Iacono (2014) conducted a phenomenological study of the perceptions of communication interaction of three residential support workers who knew one individual well (having worked with them for 2 and 15 years). The study revealed that communication with the person with PMLD comprised: ascription of meaning, attachment, touch, movement away from age-appropriateness, learning to interact, and valuing knowledge and existing skills. With regard to humor, laughing was a valued part of interactions with the person with PMLD, it was viewed as something of a leveler within interactions, as both the support staff and person with PMLD could share laughter on more of an equal footing. It was deemed a positive part of the interactions.

Support staff enjoyed seeing laughing in the person they were supporting and felt that smiles and signs of positive affect made the more negative aspects of the support worker role worthwhile. The staff also valued sharing sad times with the person with PMLD, as well as laughter, indicating that humorous exchanges are only one important component of interactions and relationship building. Interactions involved continual ascription of meaning to the behaviors of the person with PMLD. A strong emotional component was evident in the descriptions of interaction, which also involved physical touch, and built attachment between the person with PMLD and the support staff. This was reportedly somewhat at odds with the professional role of being a carer. The idea of age-appropriate interactions was critically questioned by the phenomenological accounts.

#### Humor and as an indicator of disablist attitudes and stigma

Humor was a key component in papers investigating stigma and prejudice directed toward people with intellectual disabilities. Intellectual disability was also investigated as an object of humor and consequentially an indicator of disablist attitudes and stigma. Four papers had this focus within the review. Two investigations focused on representations of people with intellectual disabilities in the media. Goggins (2010) highlighted the complexities and lack of adequate academic debate around the distinction between laughing at and laughing with people with intellectual disabilities. The study used the case of a documentary “Laughing at the disabled” (Later renamed “Down Under Mystery Tour”) to explore the challenges around this debate within media and disability studies. It tackles some of the challenges inherent in research with and on people with disabilities and engages with the idea that further work and debate around these issues is needed.

Fudge Schormans et al. (2013) in a co-researched critique of a film featuring a disabled superhero “Defendor” discuss the importance of the film for people with and without intellectual disabilities and the representations of disability therein. They highlight the importance of the film but in one section the point is made that instead of being a positive representation of disability, instead one of the authors believed it would likely lead non-disabled viewers to see his attempts to be a superhero as humorous and funny and would simply laugh at the character. This made it more challenging for this person to relate to the central character within the film and highlights the tension between having positive representations of people with disabilities and the possibility that the non-disabled majority might simply laugh at them.

Johanson-Sebera and Wilkins (2014) wrote a paper investigating the uses and implications of the term “retarded” from its original meaning as a special educational classification, to how it is used now, based on the analysis of the social media platform YouTube. Five themes for where the where and how the term was used was found. Those were (a) the traditional use of the term, (b) in humorous context, (c) to insult or criticize, (d) as a substitute for other words, and (e) as hip hop slang. Although the stigmatizing nature of term is highlighted, for the humorous context theme the word was reportedly repurposed as a positive term, akin to recent changes to the word “sick,” being slang for “great,” in Western youth culture. Although changes were made so that person first language was adopted in the 1990s in accordance with the Individuals with Disabilities Education Act, it is clear that the general use of the term remains complex and holds negative connotations and is therefore stigmatizing for those with intellectual disability. This is especially poignant when one considers that people with intellectual disabilities may not be as able to “reclaim” the word, as other marginalized populations have with related abusive terms.

Only one cross sectional UK survey by Ali et al. (2016) collected primary data on stigma and considered humor as an operationalized aspect of stigma. This investigation found that older males with moderate intellectual disabilities were more likely to report stigma (being treated differently, like children and made fun of) compared with females. Additional impairments such as sensory, mobility and speech difficulties did not correlate with reported stigma. Overall across the 229 participants approximately one third of participants with intellectual disabilities responded affirmatively to the items “people laugh at me because of the way I talk (33.19%)/look (31.88%).” The authors highlight the need to tackle stigma at both a societal and at an individual support level.

### Quality assessment of the literature

The quality of papers selected for inclusion in the review was assessed for all papers by both authors using the standard quality assessment for evaluating primary research papers (Kmet et al., 2004). Qualitative and quantitative studies were evaluated based on 10 and 14 criteria respectively, which considered design, sampling, methodology, analysis, results, rigor and trustworthiness and conclusions. For each criterion, papers were scored either 2 (good), 1 (partial fulfillment), 0 (not fulfilled) or N/A (not applicable/relevant) with the exception of the qualitative criteria “Use of verification procedure(s) to establish credibility” which was scored as 1 (fulfilled) or 0 (Not fulfilled) (For this item ETA was used as the measure of inter-rater agreement and not Spearman's rho correlation). Dividing by the total possible score resulted in a composite overall score ranging between 0 and 1 (see Appendix 2), with < 0.5 indicating limited quality, 0.5–0.7 adequate quality, 0.7–0.8 good quality, and >0.8 being indicative of strong quality. Inter-rater agreement of the ratings was within an acceptable range for both the qualitative (*N* = 10, rho = 0.791–1.00) and quantitative (*N* = 19, rho = 0.745–1.00) ratings. Following inter-rater agreement analysis, disagreements between raters were discussed until agreement was reached.

A mean score was computed for each article to provide an overall rating of quality (see Appendix 2). In addition, a mean score for each of the criteria was used to indicate the relative strengths and limitations across all 32 included studies. Overall the majority of the papers reviewed were rated as strong (*N* = 18) or good (*N* = 10) quality. Few papers were rated as adequate (*N* = 3) or limited (*N* = 1) quality. For the quantitative papers in the study none were rated as limited quality, three adequate quality, eight good quality, and eight strong quality. For the qualitative papers in the study one was of limited quality, none adequate quality, two good quality, and ten were strong quality papers. Considering mean quality criteria scores across the papers, the quantitative papers strengths lay in well described objectives, participant group descriptions, use of robust outcome measures and detail and sufficiency of results reporting. Weaknesses were evident in the lack of experimental and intervention studies, lack of control for confounding variables and lack of variance estimates (i.e., confidence intervals) presented in study findings. Due to the limited number of intervention studies, partial bias around outcome measurement and intervention description as evaluated in the Kmet quality assessment was only present in one quantitative study, Wong et al. (2015). Bias in description and recruitment of participant groups was more prevalent in the quantitative studies with four having partial bias ratings due to their inadequate description of participant groups. Similarly, for the qualitative investigations the sufficiency of objective explanation and context description, sufficient to allow transferability of findings, were strengths. Weakness included inadequacies in theoretical framework, data collection, and data analysis accounts and a lack of inclusion of reflexivity and credibility verification checks to enhance study trustworthiness. Future studies should be mindful to incorporate aspects lacking in prior studies to enhance the rigor of evidence around humor and intellectual disability. Given the limited number of relevant studies available no exclusions were made based on quality scores.

## Discussion

### Summary of main findings

After scrutinizing the extant literature, this systematic review yielded 32 papers, from which eight themes were extracted. The meanings of humor investigated characterized it as a complex interactional process, a social process, a facilitator of development, a response to social and interactional stimuli, and an inherent characteristic. This is in line with the complexity and varying conceptualizations and meanings previously assigned to humor (Moran, 2003; Coogan and Mallett, 2013). Humor was found to be a significant aspect of the social interactional lives of people with intellectual disabilities and those who provide them with support, though the extant literature reviewed was currently limited and diverse in both focus and quality.

#### The role and functions of humor in the social lives of people with intellectual disabilities

Humor comprehension and preference had not been extensively studied in the literature. The few studies that had explored this area revealed that humor comprehension can be supported by gestures. People with Williams syndrome found non-literal humor (e.g., sarcasm, irony) more difficult to understand which may impact on their social relationships. People with intellectual disabilities appreciated many various types of humor.

Research findings evident in the reviewed studies highlighted the utility and value of benevolent humor in facilitating social relationships, social closeness, carer coping and carer value, and enjoyment of the caring role. Despite this, there were few studies that specifically focused on the utility of humor in developing relationships and social closeness. Two studies highlighted the importance of shared humor for good interactions of people who do not use formal means of communication (i.e., people with PMLD). Humor was found to be an important component of online interactions for people with mild to moderate cognitive impairment and those with autism, Down syndrome and intellectual disabilities. For people with complex support needs and more severe cognitive impairments (e.g., those with Angelman syndrome), humor was also found to be a response to familiar interactional stimuli. Given the importance of humor in these contexts, it would behoove future research to consider humor as more of a key variable in interactions between people with intellectual disabilities and significant others across a variety of contexts.

Benevolent humor and sharing of social moments were key in fostering relationships, serving important social functions of humor in the lives of people with intellectual disabilities. Humor interactions, are by their very nature, complex. They can relate to laughing along together, while experiencing a shared moment (Chapman, 1983). Or perhaps, be playful, pro-social teasing or bantering, which uses fake scorn and derision to help build trust within groups or social interaction partners (Keltner et al., 2001). Humor can also be a means of trying to correct others who are deemed to be breaching social norms of a group, as satirists do to politicians (Ruch and Heintz, 2016). However, humor too can be malicious and hurtful (Billig, 2005). Mockery and ridicule serves the purpose of socially excluding the target. How we determine the intent of the humor depends on many things. At an interacting group level, it may depend on whether one is the target, the bystander/observer or the active humor protagonist. It may also depend on your general disposition or the momentary state you are in (Ruch et al., 1996).

A relationship was identified between humor and stigma. Stigma has been found to be linked to negative evaluative beliefs about the self, experiences of feeling different; with this internalizing experienced stigma negatively affecting the psychological wellbeing of people with intellectual disabilities (Dagnan and Waring, 2004). Only one study gathering primary data addressed the role of humor in stigmatizing people with intellectual disabilities, with the majority of studies gathering secondary data or involving media related case studies. A large body of more discursive literature exists focusing on critical aspects of humor and disability (e.g., Coogan and Mallett, 2013). This identifies humor as disability activism serving entertainment, societal education and re-appropriating functions (e.g., Shain, 2013). However, to date, this literature has seldom focused on humor and people with intellectual disabilities, instead primarily focusing on disability where cognitive impairment is not present.

Humor was also explored in educational settings with a focus on its role as a facilitator of development and learning. However, laughter was considered an unwanted, disruptive or inappropriate behavior in some studies too, with a small number of investigations attempting to unpick the factors which elicit laughter. Further exploration of context and differing conceptualizations of humor are clearly needed. Humor was rarely studied as a component of creativity amongst people with intellectual disabilities and autism with only one study investigating it in this way. Others highlighted the creativity inherent in the humorous expression of people with intellectual disabilities and that creativity may differ between children with and without autism. However, creativity was not always operationalized adequately within these studies.

Humor and play literature focused only on children with autism and Down syndrome and revealed that despite evidence of deficits in the social-cognitive aspects of humor, with some reductions in scope and complexity of expression, young people did demonstrate humor in their play. Although hinted at in some of the investigations of the social communication (i.e., humorous banter), there is a need for further exploration of play in adulthood in people with intellectual disabilities given its positive association with wellbeing (Proyer, 2013).

For those providing support, humor served a bonding function between carers sharing similar challenging circumstances and facilitated coping. Observed expressions of humor and joy in people with ID and shared humor between carers and those supported enabled carers to maintain a sense of satisfaction, worth and joy in their caring role, despite the difficult times they may experience.

#### Evaluation of the reviewed literature

Currently, there exists limited literature focusing on humor in the lives of people with intellectual disabilities. In the literature that does exist a range of methods have been employed. In the main, studies adopted descriptive, survey, qualitative observation or interview based methods, with a number of quasi-experimental ex post facto design investigations and very few true experiments. The quality of the reviewed papers was, in the main good, with a few exceptions, in particular the qualitative research reviewed was well conducted. Nevertheless, there were few studies providing direct empirical investigation of humor appreciation and comprehension of people with intellectual disabilities. Some studies, especially those focusing on specific syndromes, were small scale, underpowered and lacked statistical analysis, however this is understandable given the rarity of these conditions.

Within the papers included in the review, humor was often incorporated, not as a primary variable, but instead as a descriptive secondary variable or illustrative of a wider field of study (i.e., social interaction/communication) or emerged as a finding not initially sought in the study. Seldom was humor the primary variable under investigation (*N* = 6). There may be a number of reasons for this. The first relates directly to the issue of the ubiquitous nature of humor within social exchange. This common oversight is well evidenced in the humor literature (Martin, 2010). Coupled with this the difficulties recruiting and designing studies to include people with intellectual disabilities and the social and research disenfranchisement of people with intellectual disabilities may also contribute to the current lack of literature. Where humor did emerge as an important variable, it was primarily highlighted for its facilitative nature in supporting relationships, development and psychological wellbeing and because it was illustrative of positive social interactions.

### Limitations and future directions for research

Given the positive and negative impacts on wellbeing, the ubiquitousness of humor as part of the human experience and the varied conceptualizations of humor evident, there does appear to be a need for more research specifically focusing on humor and intellectual disabilities. More high-quality, primary, empirical research appears to be needed. In particular, future studies are needed in the areas of humor comprehension, representation and stigma, with greater clarity and specificity needed around the meaning and measurement of humor under scrutiny. Moreover, no study directly explored the relationship between humor and wellbeing in people with intellectual disabilities, which is a notable oversight and needs addressing in future research endeavor. Due to the potential negative effects on psychological wellbeing, the role of humor as a manifestation of societal stigma is also in need of further robust empirical investigation.

Although the search terms for this study were representative of and aligned with the review aims, other search terms may have been overlooked. This may have yielded relevant literature omitted from this review. Definitional difference in nomenclature (i.e., the term learning disabilities equating to intellectual disabilities in the UK whilst in the US and Canada it more typically equated to specific learning difficulties and developmental disabilities) made identification of papers where the participant group was people with intellectual disabilities more challenging. Alongside this, some papers did not adequately describe or define the participants which may have led to the inclusion of some papers which may not have been as directly relevant to people with intellectual disabilities (e.g., Bees, 1998; Muscott and O'Brien, 1999).

Finally, due to the novelty of the area of investigation the review presented is, by necessity, broad and multidisciplinary in scope in terms of the range of people with intellectual disabilities included. It does not focus on one specific group of people with intellectual disabilities with a range of methodologies employed in the selected studies. We aimed to explore the current state of knowledge in this field and so people with intellectual disabilities from different age groups, and their carers, were all included to provide more comprehensive and valuable insights into this unexplored area. Hence, we did not feel it appropriate to incorporate more specificity into inclusion/exclusion criteria for this initial review. Despite this, we would urge future empirical research and reviews to specify the distinct stakeholder and age groups and the particular etiology of participants. This will enable a corpus of research to be developed which can be synthesized in future meta-analysis and qualitative synthesis research. Moreover, many of the themes identified had only a handful of papers investigating them so the themes identified in this review are tentative. Further work is needed to bolster the existing evidence base and to fully explore many of the areas identified in this review. In particular the themes when developed from the review did not conform to a humor production / appreciation thematic structure as might be expected. Future research should prioritize work to better understand humor appreciation and production in people with intellectual disabilities to help achieve research parity and, more importantly, to enable more efficacious and positive support to occur through dissemination of this research work to key stakeholders and support staff. Finally, research endeavor should also be mindful to conduct humor research which is of importance to people with intellectual disabilities themselves via more inclusive and participatory strategies integrated into the research endeavor so that the work does not remain remote from the lives of people.

## Conclusions

Humor is an important aspect of the social interactional lives of people with intellectual disabilities and their carers serving important social, developmental, and emotional wellbeing functions. In particular it can serve an equalizing function in terms of interactional power fostering the experience of shared moments and building of social capital. On the other hand, humor can also be a manifestation of negative attitudes and derogation of people with intellectual disabilities, serving as a source of source of stigma and emotional harm. However, the literature as it stands is limited with the need for further methodologically robust investigations where humor is a central variable of interest. Such work will enable the ways in which humor serves both positive and negative functions in people's lives to be better understood, fostered and combatted.

## Author contributions

TP and DC contributed to the conceptualization of the review. DC was first screener of the papers focusing on people with Rett and Angelman syndrome and papers relating to carers and stigma. TP reviewed the humor in play, creativity and classroom papers, and those papers focusing on people with Down syndrome and autism. Authors shared preliminary reviewing of the core humor appreciation, comprehension and social facilitation papers. Both authors independently completed the quality reviews on all selected papers. TP and DC contributed equally to the writing of the study.

### Conflict of interest statement

The authors declare that the research was conducted in the absence of any commercial or financial relationships that could be construed as a potential conflict of interest.
