# Supplementary material for: Investigating Humor in Social Interaction in People With Intellectual Disabilities: A Systematic Review of the Literature
Source: Front Psychol. 2018 Sep 21;9:1745. doi: 10.3389/fpsyg.2018.01745 (PMC6160904; doi:10.3389/fpsyg.2018.01745)
Supplement: Supplementary file 1 [file Data_Sheet_1.docx]

# Appendix 1: Search terms employed in the review

((MH "Humour") OR TI humour* OR AB humour* OR TI humor* OR AB humor* OR TI laugh* OR AB laugh* OR Fun*)

AND

((TI ( learning N1 (disab* or difficult* or handicap*) ) OR TI ( mental* N1 (retard* or disab* or deficien* or handicap*) ) OR TI ( intellectual* N1 (disab* or impair* or handicap*) ) OR TI development* N1 disab* OR TI ( multipl* N1 (handicap* or disab*) ) OR TI "Down* syndrome" OR (MH "Developmental Disabilities") OR (MH "Intellectual Disability+") OR (MH "mentally disabled persons")) OR (AB ( learning N1 (disab* or difficult* or handicap*) ) OR AB ( mental* N1 (retard* or disab* or deficien* or handicap*) ) OR AB ( intellectual* N1 (disab* or impair* or handicap*) ) OR AB development* N1 disab* OR AB ( multipl* N1 (handicap* or disab*) ) OR AB "Down* syndrome") )
