# Supplementary material for: Investigating Humor in Social Interaction in People With Intellectual Disabilities: A Systematic Review of the Literature
Source: Front Psychol. 2018 Sep 21;9:1745. doi: 10.3389/fpsyg.2018.01745 (PMC6160904; doi:10.3389/fpsyg.2018.01745)
Supplement: Supplementary file 2 [file Data_Sheet_2.pdf]

Appendix 2: Summary of studies relating to humor and people with intellectual disabilities (see bottom of table for list of abbreviations and key for themes)

Author & year	Country	Focus	Design/Approach	Key sample features	Age range M(SD^)	% Male	Sample size	Findings	Theme	Quality Score (0-2)
Adams et al. (2015)	UK	Changes, with age, in the laughing and smiling of individuals with AS	Mixed 2 x 3 experiment, repeated on one factor.	12 participants with AS. VABS Time 1 = 12.4 (2.9), Time 2 = 32.9 (6.69)	Time 1 = 6.6 yrs (2.9), Time 2 = 10.9 yrs (3.1)	64%	12	This study compares levels of laughing and smiling longitudinally at two time points and across three within group experimental conditions [full social interaction (with eye contact), social interaction with no eye contact, proximity only]. No differences were noted in frequency of laughing and smiling over time in any condition. However, with age as a covariate, the frequency of laughing and smiling decreased over time in the full social interaction (with eye contact) condition only.	6	0.78
Ali et al. (2016)	UK	Self-reported stigma and its association with socio-demographic variables and physical disability.	Cross-section survey study. Data was collected via a checklist designed to gather information from participants about their experiences of stigma and demographic characteristics.	75% had mild ID. Sensory problem (32%); speech abnormalities (33%); mobility problems (11%); 65% had at least one health problem, including epilepsy (17%), genetic disorder (9%) or a syndrome including DS (5%).	40.9 yrs (11.4)	52%	229	This study investigated whether socio-demographic variables and physical disability (e.g. sensory impairment and mobility problems) were associated with self-reported stigma in people with ID, and examined whether age, sex and ethnicity modified the relationship between severity of intellectual disability and self-reported stigma. Older participants reported more stigmatizing experiences. Participants with moderate ID were more likely to be made fun of and treated as children. Moderate ID males reported more stigma compared to females.	8	0.8
Bees (1998)	Canada	Description of the success of the GOLD Program	Qualitative descriptive study; Program description	Over 50 adolescent students classified as gifted or having a learning disability or having unique needs	14-18yrs	92%	>50	The GOLD program was described after running for over an eight-year period. This program entailed GOLD students who had attended at least one GOLD class of three hours per week, as part of an eight-class timetable. The class taught assertiveness, self-advocacy and strategies to manage anger, panic attacks, unproductive work habits and ineffective studying via discussion and role-play. The environment reportedly encouraged hope, humor, trust, and student decision making. No empirical results formally reported. Findings appeared based on author report with no additional data gathered from users of the program to corroborate this.	3	0.35
Chadwick & Fullwood (2017)	UK & Ireland	The experience of people with ID of being online and using social media and how this relates to their sense of self, social relationships, and identity.	Semi-structured, phenomenological, qualitative interview based study	11 adults with mild to moderate ID who used the Internet and Facebook. Ages ranging 20 to 43 yrs. N=2 18.1% with DS & N=5 45.5% with ASD.	20-43 yrs; 28.5 yrs (6.6)	55%	11	Two global themes emerged. (i) Online relatedness and sharing. (ii) Online agency and support. One subtheme related to sharing online life and being connected to significant others which supported maintenance and envelopment of social capital with family and friends. One aspect of this referred to by four of the eleven participants was humor which took the forms of playing practical jokes, banter and 'taking the mick out of each other' and these interactions were viewed positively by participants as the most enjoyable online activities engaged in.	2	0.83

Degabriele & Walsh (2010)	Ireland	To determine the kinds of humor children with ID appreciate and how mode of joke presentation influences humor comprehension	Quantitative, descriptive study utilizing cross-sectional data gathered using specifically devised humor comprehension and appreciation tasks.	Participants had a diagnosis of mild (78.8%) or moderate (22.2%) ID. VMA=7;0 (2;3)..	9 yrs (1yr 7mths; Range 7–11 years)	55%	9	Findings suggested the greatest appreciation was expressed for physical and visual humor. Gestured jokes were understood better than verbally presented jokes. The greatest appreciation was expressed for physical and visual humor. Non-specific scenes (i.e. scenes with no particularly funny elements) were also rated highly. Jokes presented with gestures were understood more than jokes told without supports. These differences in comprehension, arising from supported/ unsupported jokes, were statistically significant.	1	0.79
Forster & Iacono (2008)	Australia	To explore and describe paid support staff experiences of communication with a person with PIMD	Phenomenological qualitative interview based study	3 disability support workers one had worked with the person with for 2 years and the other two had known the person with PIMD for 15 years.	Not specified	100%	3	Affect attunement incidents were observed in 16 of the pairs. The presence of affect attunement may be an indicator of interactional quality. The disability support worker's attunement behavior was in response to subtle, short duration behaviors of participants with PIMD. Laughing was a valued part of interactions with the person with PMLD, it was viewed something of a leveler within interactions as both the support staff and person with PMLD could share laughter on more of an equal footing and it became a positive part of interactions. Support staff enjoyed seeing the person they were supporting laughing and felt that smiles and signs of positive affect made the more negative aspects of the support worker role worthwhile.	7	0.80
Forster & Iacono (2014)	Australia	To explore and define the nature of affect attunement between paid support staff and adults with PIMD	Mixed methods exploratory, descriptive study. Natural interactions were videorecorded, observed and analysed for affect attunement.	21 dyads of adults with PIMD and one of their support staff. 42.9% of the adults with PIMD had CP, 33.3% had epilepsy & 23.8 reportedly had specific syndromes (DS, CRS & RS).	Adults with PIMD 37 yrs (Range = 24-55 yrs); Support staff 48 yrs (Range 38-64 yrs)	Adults with PIMD 57%; Support staff Not specified	21	Communicating in a way that was perceived to be the preferred style of the person with PIMD was important to the Disability Support Workers. Effort-attention-emotion was one of the more common eliciting behavior combinations observed. The presence of affect attunement was identified as an indicator of interactional quality, which includes expressions of emotional happiness and attempts to share humor during interactions. The study highlights the natural communicative skills some support staff possess, the inter subjectivity of dyadic interactions and demonstrates a way two people can share through very subtle interactions regardless of communication impairment.	2	0.80
Fudge-Schormans et al. (2013)	Canada	To consider the representations of ID within film and the importance of these representations for people with and without ID	Inclusive group work resulting in a discursive critique of a media representation	Participants were made up of an inclusive research group which included three people with ID, two academic researchers, and four graduate-level university students.	Not specified	Not specified	Not specified	The reported outcome was that this film was important for people without ID to see. Not only did the film show how those with ID are devalued, it also showed people with ID as capable, heroic, brave, and smarter than people think. The possibility of mockery of characters with ID in film highlighted the tension between positive representations and stigma and may make protagonists with ID less relatable for people with ID.	8	0.88
Gagić et al. (2015)	Serbia	To determine the difference in creative abilities of children with Mild ID prior to & following visual prompting during the creation of an art drawing	Quantitative quasi-experimental pre-post design without a control group. The Urban-Jellen test was used to assess creativity (which incorporates humour alongside completion, material manipulation and non-stereotypical use of elements)	Children with mild ID (IQ range 50 to 69 based on WISC). Absence of additional conditions and impairments (i.e. neurological, psychiatric, sensory, severe emotional & multiple disorders).	Age 8 to 16 years (M=11.75, SD=2.12).	55.1%	69	Statistically significant differences were found pre and post visual prompting in aspects of creativity (including humor). The authors highlight the importance of visual incentive for the expression of children's creativity.	4	

Godbee & Porter (2013)	Australia	To compare the comprehension of sarcasm, metaphor and simile in WS relative to both TDCA and TDMA	Quantitative quasi-experimental ex post-facto design study, gathering observational and self-report data.	Twenty-six participants with WS (IQ M = 46.9; SD=18.1); 26 TDCA; and 26 TDMA.	Not specified	WS = 46%	78	Comprehension of non-literal language in WS was significantly below TDCA levels, but was not significantly different to TDMA levels. For typically developing controls, each of the cognitive measures was strongly correlated with each of the measures of non-literal language comprehension. The same relationships were not always found for participants with WS. In particular, sarcasm comprehension in WS was not significantly correlated with any of the assessed cognitive abilities, and expressive vocabulary was not significantly correlated with any measure of non-literal comprehension.	1	0.87
Goggins (2010)	Australia	To explore the place of disability in Australian culture, the role of power, questions of ethics and the cultural politics of disability in education.	A qualitative case study analysing secondary data regarding 'Laughing at/with the Disabled' a film and research project comprising a humorous story, starring people with ID.	The film, the public record covering the criticisms of the research project involving, disciplinary action by Queensland University of Technology, and responses by those involved in the project formed the corpus of data for the study.	Not specified	Not specifi ed	Not applicabl e	The film starred two people with ID. From the findings of this study, it was concluded that the issue of laughing at versus laughing with people with ID is complex and emotional responses may impede adequate academic debate around this issue. The film reportedly offered new visions, scripts, challenges, and ideas about the cultural politics of disability and education.	8	0.75
Griffiths & Smith (2016)	Ireland	Investigates how people with severe and PMLD interact with those with whom they come into contact and how people without intellectual disability interact with those with severe and PMLD.	An observational qualitative study using a grounded theory approach.	Two male adults with PMLD, one 18 yrs with epilepsy and one 26 yrs with quadriplegic CP. Neither had recognizable spoken words, both could vocalize and one used gesture. Two female paid carers who had known their communication partner ≥ 1 year.	Adults with PMLD 22 yrs; Paid carers age not specified	Adults with PMLD 100%; Carers 0%	2; 0	Three dyads of people with PMLD were observed though only 2 were analyzed as saturation was reached prior to analyzing the third. Attuning was identified as a central process that calibrates and regulates communication. Within this theory, humor is described as an indicator of empathic harmony and pro attunement and a manifestation of: (i) close psychological contact via a smile; (ii) shared amusement via a smile or laughter.	2	0.80
Griffiths & Smith (2017)	Ireland	Explores the transactional nature of interaction between people with severe or PMLD and those providing them with support.	The article utilized qualitative observational data and conducted a narrative analysis of interactions between two dyads.	Two dyads each comprising a person with severe or PMLD and a service worker. Dyad 1 consisted of J an 18-year-old man and his paid carer, who had known him for 1 year. Dyad two consisted of a 26-year-old man and his nurse keyworker who had known him for over a year.	Adults with PMLD 22 yrs; Paid carers age not specified	Adults with PMLD 100%; Carers 0%	2; 0	Attuning was identified as a central process that calibrated and regulated communication. Attuning was conceptualized as a bidirectional, dyadic communication process. Understanding this process may support more effective communication between people with severe or profound intellectual and multiple disability and their interaction partners. This partner paper to Griffiths and Smith (2014) also reports humor as an important part of positive attuning.	2	0.88

Hobson et al. (2009)	UK	Investigated whether there is a difference in qualities of spontaneous and modeled playful engagement (incorporating playful pretend & mechanics of pretend play) between children with ASD and children with ID.	A quantitative quasi-experimental study utilizing a mixed design comparing aspects of pretend play across two play conditions (modeled and spontaneous) and across two groups (children with ASD and children with developmental delay/learning difficulties matched on VMA).	(i) 16 children (all boys) with ASD (CA Mean = 9.6(2.11), VMA Mean = 5.3(1.11)) and (ii) 16 children (11 boys, 5 girls) with learning difficulties or developmental delays but without ASD (CA Mean = 10.4(1.6), VMA Mean = 5.0(1.2)).	Children with ASD 9.6 years (2.11) Comparison group = 10.4 years (1.6)	Children with ASD 100%; Comparison group 69%	32	The two groups were similar in the mechanics of play, for example in making one thing stand for another and using materials flexibly. Differences were found for the children with ASD who showed less playful pretend, for example investment in the symbolic meanings given to play materials, creativity, and fun. Playful pretend and the mechanics of pretend play did not differ across the two play conditions.	5	0.79
Hudenko et al. (2009)	USA	Tested the hypothesis that during social interactions, children diagnosed with autism would exhibit less extreme laugh acoustics than their non-autistic peers.	A quantitative descriptive and quasi-experimental ex post-facto design study comparing observed vocal expressions of emotion in children with ASD and control participants. Laughter, recorded during a series of playful interactions with an examiner was compared.	15 eight to ten-year-old children diagnosed with ASD (13 male, 2 female); Two sex matched control groups: (i) 15 TDCA ; (CA, ± 3 months of participant with autism); and (ii) 15 TDMA M=6.9(2.1) (VMA equivalent ± 3 mths of participants with ASD M=6.8(2.1).	Children with ASD 9.1yrs (.77), Comparison group (i) 5.7yrs (1.8); Comparison group (ii) 9.0yrs (.70).	87% for all three groups	45	The voiced laugh contains a fundamental frequency (F0). Results showed that children with ASD exhibited only one type of laughter (voiced), whereas comparison participants exhibited two types (voiced and unvoiced). No group differences were found for laugh duration, mean fundamental frequency (F0) values, change in F0, or number of laughs per bout. Findings are interpreted to suggest that children with autism express laughter primarily in response to positive internal states, rather than using laughter to negotiate social interactions.	5	0.77
Johanson-Sebera & Wilkins (2014)	USA	To discover how the word retarded is currently used in popular culture in the USA	Qualitative descriptive thematic analysis of online youtube videos.	100 Youtube videos with the word "retarded" in the title randomly selected from 222 videos that met inclusion criteria.	None	None	0	Five themes emerged: (a) the traditional use of the term retarded, (b) the use of retarded in humorous context, (c) the use of retarded to insult or criticize, (d) the term retarded as a substitute for other words, and (e) the slang use of retarded in a hip hop context. The simplistic rules for the complexities of language were explored as a conclusion.	8	0.78
Johnson et al. (2012)	Australia	To (i) identify social interactions between the people with severe intellectual disability and those with whom they have positive relationships and (ii) detail the nature of those interactions.	Constructivist, grounded theory approach analyzing qualitative observational and interview data.	Adults with severe intellectual disabilities and significant others, identified as someone who they have a pleasurable relationship with.	29.3 yrs	66%	6	Fourteen factors were found which broke down into five levels. The highest order was “sharing the moment”, this had two factors: (1) having fun and (2) hanging out. Having fun was broken down further to a second level that was routines and comedy. Hanging out second level comrpised contact and presence. Level three extraction for routines incorporated rhythmic play, games and songs, and mimicry. Level three factors included under comedy were vulgarity and pranks, under vulgarity jesting was extracted and under pranks banter was identified as a subtheme. From contact and presence no further levels were extracted. Humor and mirth were identified as key components of positive relationships with people with severe ID.	2	0.85

Jones & Goble (2012)	USA	To determine the factors which made a mentoring program to enhance education and social experiences effective and how the current program should evolve.	An evaluative, qualitative research study interviewing key stakeholders in the mentoring program	Participants included 4 university professors (6–19 yrs of experience), 4 students with ID (age range 19–21 yrs), 3 parents (mothers of the 3 students with ID), 1 representative from the university's disability service (3yrs experience), and 12 mentors (aged 20-22yrs).	Students 20 yrs; Mentors 21 yrs	Not specified	7	This study found ways for students with ID to be successfully supported on one university campus, generating new ideas for improving the quality of those mentoring supports. Prioritising spontaneous fun and socializing was highlighted as nurturing the development of an equal and reciprocal relationship and as a key component for creating and improving effective mentoring partnerships in University settings.	3	0.90
Krishan, Bachelor and Porter (2017)	Australia	To investigate humor comprehension and the use of mental state language in individuals with WS and DS and compare these with TDCA and TDMA control groups.	A quantitative, quasi-experimental ex post facto design comparing humor comprehension across five groups: (i) Children with WS; (ii) Children with DS; & (iii) TDCA, and those matched for MA with the participants with (iv) WS and (v) DS..	Children: (i) 27 with WS (Mental age ranged from 3.58- 9.33 years, M=5.57, (1.35)).16 with (ii) DS (Mental age, ranged from 4- 6.16 years, M=4.87, SD=0.75); (iii) 27 neuro-typical (13 females) age matched adults; and 30 Mental Age control participants, (iv) 17 WS (Mean MA = 6.11 (1.46); (v). I 13 DS (Mean MA 5.17 (0.61).	Group (i) WS 14.9 yrs (7.0); (ii) DS 14.5 (6.4); (iii) TDCA 15.2,(6.8); (iv) TDMA-WS 6.1 (1.4); (v) TDMA-DS 5.1 (0.6)	Participant group: WS 52%; DS 63%; TDCA 52% TDMA-WS 47.1; TDMA-DS 38.5	100	The study depicting 23 cartoons from which participants had to say what was funny. Findings reveal that compared to the TDCA group lower levels of humor comprehension were evident in participants with WS and DS but these two groups did not differ from their TDMA control groups. Fewer physical emotion terms such as laughing, crying and screaming were evident in the participants with WS compared with the TDCA and the participants with DS but were equivalent with TDMA group. The use of cognitive words, such as thinking showed a difference with both the participants with WS and DS using fewer than TDCA controls, but they performed similarly to each other and to their relative TDMA. The importance of intellectual ability in processing humor is highlighted.	1	0.87
MacDonald et al. (2007)	UK	Aimed to identify patterns of respite use and coping strategies among family caregivers in Ireland	A cross-sectional quantitative survey. Data was collected via a checklist designed for the study and an existing measure of coping strategies.	Purposive sample of carers with family member with ID at home. From large database. A total of 46 children with ID and 71 carers. This was a 43% response rate from the identified sampling frame	Not specified	Not specified	N=117. 46 children with ID and 71 carers.	The likelihood of families using respite care was not significantly related to presence of challenging behaviors or level of support required by children. Also, not significantly related to family size and social support. With regard coping strategies, a high proportion of carers identified 'keeping a little free time for myself' as a helpful strategy to manage stress. The results also indicated that a high proportion of carers derived considerable help from managing meaning coping strategies such as 'seeing the funny side of the situation' and 'realizing that the person I care for is not to blame for the way they are', particularly male carers. Female carers tended to use 'problem solving' strategies, establishing routines, finding information and trying out solutions until one works. Hence maintenance of a sense of humor was a key coping strategy used.	7	0.73

Messier et al. (2008)	Canada	To describe the play performance of children with ID at school entry, and to investigate the association between degree of intellectual impairment and play performance.	Quantitative, descriptive observational study of play performance, characterized relative to developmental level, along with interests, abilities and attitudes of play. Play performance was assessed using the ALB and the PPS-R.	School age children age from 5 to 7 with IQ <70.	6.8 yrs (±7mths)	55%	27	The results highlight the strengths and limitations in play behaviors of children with ID. Children demonstrated good abilities in use of objects and space in both assessments. Furthermore, four out of six elements of the Ludic attitude: curiosity, initiative, pleasure, and spontaneity were present irrespective of IQ level. However, sense of humor and enjoyment of challenge were less present. Interestingly, the imitation dimension showed relative weakness suggesting that this learning method may not be optimal in the school setting.	3	0.73
Mount et al. (2011)	UK	To investigate social behaviors amongst people with AS in different contexts	Quantitative, experimental within groups study investigating social behaviors shown by individuals with AS (the amount of laughing/ smiling, looking, reaching, touching and pulling) across three levels of social interactional context (adult familiarity, eye contact and talking),	Seven female and eight male participants aged between 3 and 18 years all diagnosed with AS. Thirteen had the genetic cause being deletion, one imprinting defect and one uniparental disomy. Complete data with primary carers was available for 11 participants who were included in the analysis. Age-equivalent adaptive level 13.1 mths (4.1).	7.8 (4.3)	63.6%	11	Overall, there was more adult laugh/smile in the eye contact and talking conditions. Considerable variability was seen in participants for frequency of participant's behaviors across the experimental sessions. The most variable was for laughter/smiling. No statistically significant effects of the factors were evident for participant laugh/smile and eye contact. However, significantly, more approach behavior was shown by participants when the familiar adult engaged in eye contact compared with when the unfamiliar adult engaged in eye contact.	6	0.91
Muscott & O'Brien (1999)	USA	To examine participants' knowledge of the curriculum and perceptions of a Citizenship program	Ethnographic qualitative study with data gathered via interviews.	21 were male and 11 female participants were children attending an inner-city elementary school (Kindergarten to 6-7yrs). Of the 32 total students, eight had no disability or were gifted, and the remaining 24 had identified disabilities or were at-risk of failure at school.	Not specified	52%	32	Participants found learning about character to be rewarding and fun. Overall the children with behavioral and learning disabilities were extremely satisfied with the program, they perceived it to be fun, friendship, teamwork and cooperation and learning. Many students thought the program taught them, how to cooperate with students of different ages, to solve problems, what it means to be a citizen-leader and a team member, how to be responsible when confronted with the answers to a test, and how to reach out to peers from diverse backgrounds who are new to the school.	3	0.91
Oliver et al. (2002)	UK/ Greece	To examine environmental interactional influences on the social behavior of people with AS	Quantitative within participant case series assessing the occurrence of smiling and laughing behavior in relation to social stimuli (4 conditions: alone, proximity, instruction & interaction)	3 children with AS (2 females aged 7yrs (MA 14 mths) and 11yrs (MA 12 mths) and 1 male age 15yrs (MA 20 mths).	11 yrs	33.3%	3	Both smiling and laughing occurred least in the alone condition, followed by the proximity condition and then the instruction condition both of which had low to moderate laughing and smiling. The interaction condition was observed with the greatest amount of laughing and smiling for all three children. Findings did not support the assertion of inappropriate and unelicited smiling and laughing in children with AS and indicates that the environment plays a significant role in eliciting smiling and laughing behavior.	6	0.73

Reddy, Williams, & Vaughan (2002)	UK	To describe the humor and laughter in the everyday lives of children with ASD and DS	Quantitative descriptive and quasi-experimental, ex post facto design study comparing children with ASD and children with DS. Data was gathered qualitatively and quantitatively via parental accounts and video taped observations	19 children with ASD and 16 children with DS. No detailed information was given regarding the parent participants.	Children with ASD 49.6 (9.0) mths Children with DS 41.3 (12.2) mths	68.6%	35	Parental Reports: No group differences were reported by parents in the frequency of laughter between children with ASD and DS. Differences in what the two groups laughed at were reported though. Both groups enjoying tactile events (including tickling rough and tumble play and chasing and more subtle tactile contact), visual events or auditory events, though children with AS laughed less at the latter two sensory events. Significantly more children with DS laughed at socially inappropriate acts and funny faces and children with ASD laughed significantly more for no apparent reason. Children with DS were reportedly more likely to engage in clowning and teasing. Observational Data: Laughter where the content was not shared was observed significantly more in the children with ASD. Shared laughter at an external target, reacting to the laughter of others and responding to laughter directed at the child were all observed significantly more in children with DS. Total laughs, solitary laughs and laughs in response to the behavior of an interactional partner did not significantly differ between the two groups.	5	0.64
Sandhu et al. (2012)	UK	To explore the emotional challenges faced by staff working in a sex offender treatment program for people with intellectual disability	Semi-structured, phenomenological, qualitative interview based study	8 staff members working in a sex offender treatment programme. Working between 1 and >10 years in the program.	Aged 20-50 years	62.5%	8	Findings suggest complex individual and shared responses of working with sec offenders with ID with challenges about how to empathise with this group of people who are also offenders and ongoing experiences of stress and anxiety. Coping and emotional defences were used by participants, this included shared humor being reported as one of the most important ways of dealing with the negative emotions experienced as part of this role. However, use of humor could also be used as a defence mechanism to prevent exploration of the emotional and personal impact of this type of support work.	7	0.85
Schieltz et al. (2011)	USA	The purpose was to evaluate the effects of FCT on non-targeted destructive and disruptive behavior.	This within participant case series involved identification of specific destructive responses (e.g., self injury) and a specific disruptive (e.g., stereotypy) responses, identified by the parent and shown to occur in the demand context of a functional analysis.	8 male and 2 female children. These 10 participants were preschool-aged children with DD who engaged in both destructive (property destruction, aggression, self-injury) and disruptive (hand flapping, spinning in circles, shrill laughter, screaming, crying) behaviors.	2.8 years	80%	10	A 71% reduction was observed between the initial and final baseline conditions for destructive behavior, and a 90% reduction was observed for non-targeted disruptive behavior. Both destructive and non-targeted disruptive behavior occurred at lower levels during all FCT conditions, representing a 94% overall decrease in destructive and non-targeted disruptive behavior when compared to the functional analysis demand condition. Similarly, across all FCT conditions, a 95% overall decrease in destructive behavior and an 84% overall decrease in non-targeted disruptive behavior (including shrill laughter) were observed across children when compared to the initial baseline condition.	6	0.71

Schnitzer et al. (2007)	Belgium	To evaluate an interactive, cognitive socio-emotional intervention model underpinned by Feuerstein's Instrumental Enrichment program (A classroom curriculum designed to enhance cognitive functions necessary for learning).	The intervention was evaluated via (i) A pre-post quasi experimental design with a control group. Groups were matching on the following variables: education in a school for children with learning difficulties and behavior problems, age, IQ, and sex; and (ii) Semi-structured group interviews.	24 pupils constituting the intervention and 24 pupils constituting the control group. Two classes with the highest rates of behavioral problems were purposively selected to be in the intervention condition. There were 16 boys in each group.	Age ranged between 11 and 13 years	67%	48	A marked increase in some cognitive functions (hypothetical thinking, perception and understanding of humor) was found between pre and post measures for the intervention group. Differences between experimental and control groups were not statistically significant for all cognitive functions and social-cognitive processes. Children in the intervention group showed a significantly higher understanding of 'complex humor'. Quantitative accounts from class 1 intervention participants did not feel that the intervention was effective, however, the accounts of the personal changes of class 2's participants confirmed that there were positive outcomes from the programs. Positive changes were not always attributed to the program.	3	0.82
Short et al. (1993)	USA	To investigate differences in the comprehension, production and appreciation of humor between students with and without disabilities.	Quantitative quasi-experimental ex post facto design gathering data via observation and self-report measures. Specifically designed humor stimuli were presented to participant groups.	Three student groups participated: (i) neurotypic (N=41, 20 2nd graders (M IQ = 106.4), and 21 4th graders who were typically achieving) (M IQ = 104); (ii) 14 fourth graders with learning disabilities (M IQ = 90); and (iii) 12 fourth graders who had developmental disabilities (M IQ 71.1 (8.1)).	(i) 2nd graders 8.1yrs, 4th graders 10.3yrs; (ii) Those with LD 10.4yrs; (iii) DD 10.9yrs. 76% of the sample were Black.	42% Overall. (i) 2nd graders 40%, 4th graders 27%; (ii) LD 58%; (iii) DD 42%	67	Humor appreciation, comprehension and production scores were positively and moderately related. Humor production and comprehension related moderately to intellectual ability. Humor comprehension was lower for the groups with disabilities. No differences in humor production were observed between those with learning disability and neurotypic students. Developmental disabled participants lacked sensitivity in humor appreciation. The appreciation measures mirth and funniness related to funny and neutral cartoons but only minimally related to intellectual ability. Group differences were found for comprehension of humor, which mirrored development (highest comprehension for normally achieving 4th graders, followed by the 2nd graders). Comprehension for both the children with developmental handicaps and those with learning disabilities were lower than the typically achieving children, with those children with developmental disabilities having the lowest humor comprehension.	1	0.87
St. James & Tager-Flusberg (1994)	USA	To investigated examples of naturalistic humor in children with ASD and DS	Quantitative, quasi-experimental ex post facto design gathering observational data from (i) children with ASD; and (ii) 6 age- and language-matched children with DS. Participants were videotaped over the course of 1 year. Humor episodes were analyzed on three dimensions: cognitive developmental, social, and intentionality.	Six children with ASD and 6 with DS aged between 3 and 6 years old	Children with ASD 5.6 yrs, Children with DS 5.4 yrs	Group with ASD 100%; Group with DS 66%	12	The children with ASD produced significantly less humor overall (165 humor episodes vs. 547 from children with DS. The amount of humor produced significantly increased with age for both the children with ASD and DS but did not vary significantly with IQ and language levels. Children with ASD produced verbal incongruity humor aligned to typical development of humor. The only jokes in the study were told by two of the children with DS, these were riddles. The children with ASD produced and appreciated humor to a limited extent in a naturalistic setting, at a significantly reduced level compared to matched controls with DS. Children with ASD appreciated and produced less humor involving nonverbal incongruity. Within group and within child variability in type of humor produced was more prevalent in children with ASD. Most humor (80%) produced by both groups was at a non-verbal level, with 125 out of 712 humor episodes being verbal incongruity, of which 100 were produced by participants with DS. With regard to initiation of humor interactions, 12% of all humor episodes were initiated by mothers, 75% by children and 13% as a joint interaction. Mothers initiated more for the children with ASD (21% vs. 6% for the children with DS). Finally in the intentionality dimension, children with DS produced humor at all 3 levels of intentionality and all except one child with ASD also produced humor at all three levels.	5	0.82

Sullivan, Winner & Tager-Flusberg (2003)	USA	To discern the ability of adolescents with ID to distinguish lies from ironic jokes	Quantitative, quasi-experimental ex post facto design gathering self-report data from three participant groups with ID.	Three clinically diagnosed groups of adolescents: (i) with Williams syndrome (N=16; IQ M=65.7 (12.2)); (ii) with Prader-Willi syndrome (N=11; IQ M=75.1 (12.7)); and (iii) non-specific ID (N=12; IQ M=66.5 (5.7)).	WS Group = 12.3 (SD 1.1) years PWS Group = 12.8 (SD 2.9) years NMR Group = 12.0 (SD 2.7) years	WS Group = 44% PWS Group = 82% NMR Group = 42%	39	When deciding the form of the nonliteral utterance and justifying their responses, almost none of the participants in any of the groups were found to be able to correctly classify the ironic jokes. Instead they judged ironic jokes to be lies because they did not correspond to reality. For the joke stories only, adolescents with WS provided significantly more reality justifications than did adolescents with PWS and MRU adolescents suggesting that despite the lack of group differences on the lie/joke discrimination question the adolescents with PWS and MRU focused more on the mental states of the story characters, whereas the adolescents with WS justified their lie/joke decisions by judging the statement against what really happened.	1	0.86
Wong et al. (2015)	Australia	To determine the prevalence of sleep problems in Rett syndrome and investigate determinants of the trajectories of sleep problems, including age, MECP2 mutation and use of treatment.	Quantitative, within group longitudinal study utilizing data from the Australian Rett Syndrom Database Data were collected at six time points over 12 years from 320 families.	People with Rett syndrome whose families were registered with the Australian Rett Syndrome Database.	Age ranged between 2 and 35.8 yrs (SD missing)	Not specified	320	Over the six time points where data was collected, the prevalence of the sleep problems night waking and laughing reduced with age, ranged from 35% in 2011 to 55% in 2006. Similarly, for night waking and screaming, the prevalence was 26% in 2011 and 36% in 2006. The prevalence of any sleep problem was generally very high (>91%, except 85% in 2002) in the 7 years or younger age group and moderately high (73–98%) in the older age groups. In those aged ≥_18 yrs the conditions appeared to be slightly less common. For night laughing, 60–88% of girls in the youngest age group experienced the problem. Two developmental trajectories were identified, labeled as 'high baseline' and 'low baseline', reflecting the relative estimated level of prevalence of sleep problem at the first observation period in each group. The prevalence of receiving any treatment for sleep problems across the six time points was, on average, 16.1%.	6	0.73

[Key for themes: 1 Humor Comprehension and Appreciation among people with intellectual disabilities; 2 Humor, Social Facilitation and Social Capital; 3 Classroom humor and laughter; 4 Humor and Creativity; 5 Play, Humor and Laughter in Children with Autism and Down Syndrome; 6 Laughter as disruptive, unelicited or inappropriate behavior; 7 Humor as a Coping Strategy for Carers & Support Staff; 8 Humor and as an indicator of disablist attitudes and stigma]

[Abbreviations: ALB Assessment of Ludic Behavior; AS Angelman syndrome; ASD Autistic Spectrum Disorder; DD Developmental Disabilities; DS Down syndrome; CA Chronological age; CP Cerebral Palsy; CRS Congenital Rubella syndrome; FCT Functional Communication training; ID Intellectual Disabilities; IRR Inter rater reliability; ns statistically non-significant; PIMD Profound Intellectual and Multiple Disabilities; PMLD Profound and Multiple Learning Disabilities; PPS-R Preschool Play Scale – Revised; RS Rett syndrome; TDCA typically developing chronological age-matched controls; TDMA typically developing controls matched on mental age; VABS Vineland Adaptive Behavior Scales; WISC Weschler Intelligence Scale for Children; WS William syndrome; VMA Verbal mental age; yrs Years]

^ Where provided or calculable from the data provided in the paper.
